# Recent developments in multi-omics and breeding strategies for abiotic stress tolerance in maize (*Zea mays* L.)

**DOI:** 10.3389/fpls.2022.965878

**Published:** 2022-09-23

**Authors:** Muhammad Qudrat Ullah Farooqi, Ghazala Nawaz, Shabir Hussain Wani, Jeet Ram Choudhary, Maneet Rana, Rameswar Prasad Sah, Muhammad Afzal, Zahra Zahra, Showkat Ahmad Ganie, Ali Razzaq, Vincent Pamugas Reyes, Eman A. Mahmoud, Hosam O. Elansary, Tarek K. Zin El-Abedin, Kadambot H. M. Siddique

**Affiliations:** ^1^The UWA Institute of Agriculture, University of Western Australia, Perth, WA, Australia; ^2^Department of Botanical and Environmental Sciences, Kohat University of Science and Technology, Kohat, Pakistan; ^3^Mountain Research Centre for Field Crops, Sher-e-Kashmir University of Agricultural Sciences and Technology of Kashmir, Srinagar, India; ^4^Division of Genetics, Indian Agricultural Research Institute, New Delhi, India; ^5^Division of Crop Improvement, ICAR-Indian Grassland and Fodder Research Institute, Jhansi, India; ^6^Division of Crop Improvement, ICAR-National Rice Research Institute, Cuttack, India; ^7^College of Food and Agricultural Sciences, King Saud University, Riyadh, Saudi Arabia; ^8^Department of Civil and Environmental Engineering, University of California, Irvine, Irvine, CA, United States; ^9^Department of Biotechnology, Visva-Bharati, Santiniketan, India; ^10^Agronomy Department, University of Florida, Gainesville, FL, United States; ^11^Graduate School of Bioagricultural Sciences, Nagoya University, Chikusa, Japan; ^12^Department of Food Industries, Faculty of Agriculture, Damietta University, Damietta, Egypt; ^13^Plant Production Department, College of Food and Agriculture Sciences, King Saud University, Riyadh, Saudi Arabia; ^14^Floriculture, Ornamental Horticulture, and Garden Design Department, Faculty of Agriculture (El-Shatby), Alexandria University, Alexandria, Egypt; ^15^Department of Geography, Environmental Management, and Energy Studies, University of Johannesburg, Johannesburg, South Africa; ^16^Department of Agriculture & Biosystems Engineering, Faculty of Agriculture (El-Shatby), Alexandria University, Alexandria, Egypt

**Keywords:** genomics, miRNA, genome editing, phenomics, transcriptomics

## Abstract

High-throughput sequencing technologies (HSTs) have revolutionized crop breeding. The advent of these technologies has enabled the identification of beneficial quantitative trait loci (QTL), genes, and alleles for crop improvement. Climate change have made a significant effect on the global maize yield. To date, the well-known omic approaches such as genomics, transcriptomics, proteomics, and metabolomics are being incorporated in maize breeding studies. These approaches have identified novel biological markers that are being utilized for maize improvement against various abiotic stresses. This review discusses the current information on the morpho-physiological and molecular mechanism of abiotic stress tolerance in maize. The utilization of omics approaches to improve abiotic stress tolerance in maize is highlighted. As compared to single approach, the integration of multi-omics offers a great potential in addressing the challenges of abiotic stresses of maize productivity.

## Introduction

**FIGURE 1:**
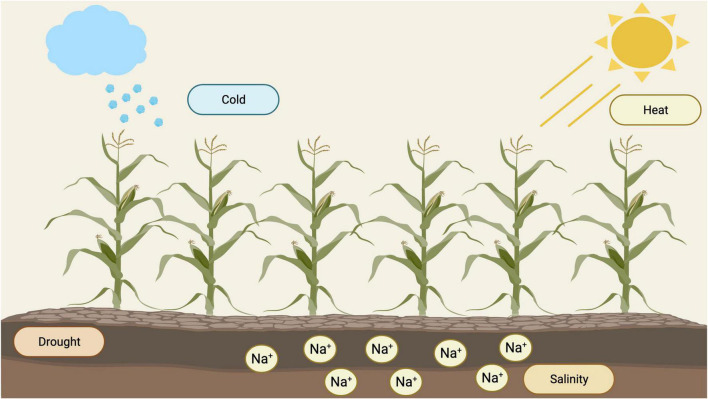
Types of abiotic stresses that affect yield productivity in maize (https://biorender.com; accessed on 21 February 2022).

Maize (*Zea mays* L.) is one of the most cultivated crops across the globe for food, animal feed, and as a source of biofuel (Ranum et al., 2014; Molla et al., 2019; Choudhary et al., 2020). Maize yield is highly dependent on a broad spectrum of climatic and soil conditions. However, abiotic stresses such as drought, salinity, high temperature, and cold, are restricting factors that affect its yield productivity (Wani et al., 2016; Figure 1).

To date, drought is considered as a significant threat to crop growth depending on its severity and duration (Chen et al., 2012; Ganie and Ahammed, 2021). Edmeades et al. (2015) reported that maize is vulnerable to drought from flowering through to grain filling stage. It also directly affects the rate of photosynthesis activity within chloroplasts. In leaves, drought stress disturbs the concentration of abscisic acid (ABA), increasing various antioxidant enzymes such as GR (glutathione reductase), APX (ascorbate peroxidase), CAT (catalase), and SOD (superoxide dismutase) (Jiang, 2002; Mehla et al., 2017). On the other hand, salinity stress affects plants under irrigated and non-irrigated situations. A significant proportion of irrigated (50%) and all cultivated (20%) land is under salinity stress (Wang et al., 2017). This affects the growth and development of maize; however, the response of plants varies based on the degree of salinity and crop growth stage (Farooq et al., 2015). Short-term exposure of maize plants to salt stress influences its growth owing to osmotic stress. In salt-affected soils, excessive buildup of sodium and chloride ions in the rhizosphere leads to severe nutritional imbalances in maize due to strong interference of these ions with other essential mineral elements. Another type of stress, known as heat stress, reduces crop growth and productivity (Wahid et al., 2007; Fahad et al., 2017; El Sabagh et al., 2021). This affects cell metabolic activity and signals physiological networks, resulting in poor pollen dehiscence and fertility, stigma and silk emergence, seed set, and grain filling, reducing maize grain yield (Barnabás et al., 2007). Maize plants are also sensitive to low temperatures (<15°C) and can kill imbibed seeds and induce leaf senescence in maize (Miedema, 1982; Foyer et al., 2002). Additionally, chilling temperature (10°C) combined with excessive light stress reduces CO2 assimilation, leading to irreversible photosynthesis inhibition and cellular damage (Farooqi et al., 2016).

To withstand these abiotic stresses, plants can seize their growth activity under severe circumstances and develop various internal defense mechanisms (molecular, cellular, metabolic, and physiological) (Atif et al., 2019). For example, phenotypic stress adaptations have been observed in maize, such as reduced leaf angles, increased leaf wax, compacted tassels, and reduced evaporation rate in anthers which is crucial to preventing anther dehiscence (Shah et al., 2011). Similarly, genetic and metabolic networks are regulated in response to various abiotic stresses (Menezes-Benavente et al., 2004; Osmolovskaya et al., 2018).

**FIGURE 2:**
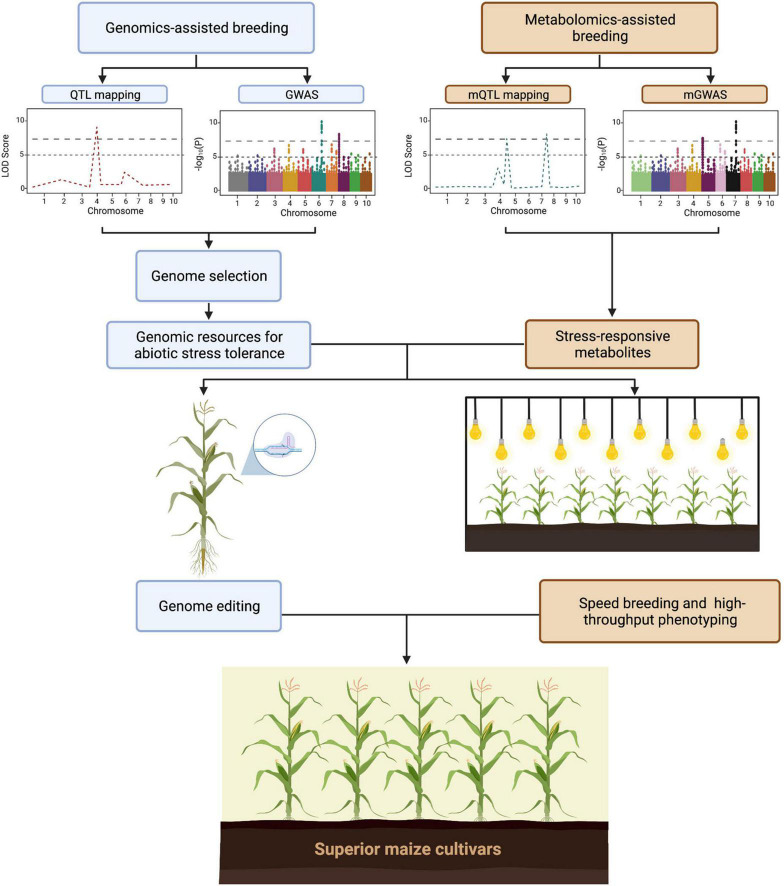
General workflow on the utilization of omics technologies in development of superior maize cultivars (https://biorender.com; accessed on 21 February 2022).

The advances in genetics and molecular biology have led to the development of high-throughput sequencing technology. As a result, plant scientists were able to identify genes and genetic regions that are associated with traits of interest. Over the years, these genes and genetic information have been successfully used for crop improvement in terms of yield, biotic stresses, and abiotic stresses (Jena and Mackill, 2008; Angeles-Shim et al., 2020; Reyes et al., 2021a). Multi-omics approaches have aided in the understanding of maize crop growth, senescence, yield, and responses to biotic and abiotic stresses (Jeyasri et al., 2021; Kaur et al., 2021). As shown in Figure 2, omics approaches such as genomics and metabolomics are being utilized to identify loci and metabolite markers that are associated with abiotic stress tolerance. In addition to this, several approaches such as genome editing, and speed breeding can be applied to hasten the development of superior maize cultivars. To date several studies have utilized omics approaches for abiotic stress tolerance. For example, phenomics have aided in the development of non-destructive phenotyping approaches. Similarly, identification of regulatory networks that are associated with abiotic stress response are now possible due to transcriptomics. Collectively, omics approaches are beneficial in plant science research specially for understanding the genome, transcriptome, metabolome, and phenome of crops.

In this review, a holistic approach was used to discuss the recent developments on abiotic stress tolerance in maize. Firstly, we discussed the morphological, physiological, and molecular responses of maize to abiotic stresses. Then, we presented studies that used conventional and marker-assisted breeding approaches to understand the genetic mechanisms involved in stress adaptation were also. Lastly, we highlighted the use of “omic” tools and utilization of wild maize relatives for genetic resource development of stress tolerance in maize.

## Physiology of abiotic stress tolerance in maize

**FIGURE 3:**
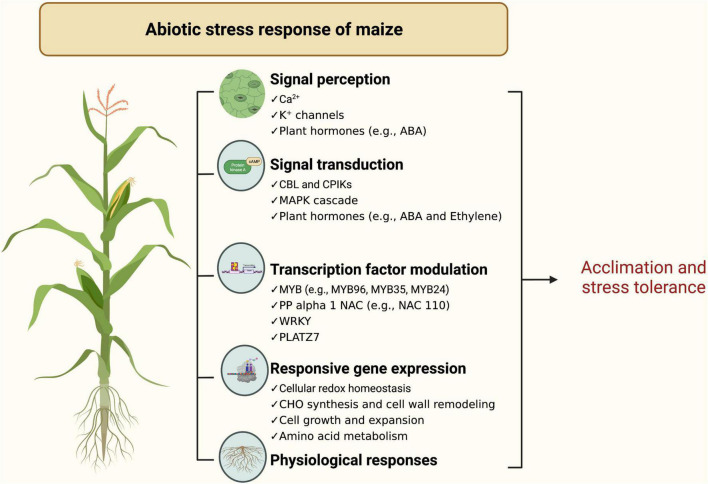
Schematic diagram of maize physiological and genetic response to abiotic stress (https://biorender.com; accessed on 21 February 2022).

Maize has various physiological responses to abiotic stresses (Figure 3). In general, abiotic stresses such as high temperature, salt, and drought alter many physiological traits such as membrane permeability, net photosynthesis, osmolyte accumulation, respiration, osmotic potential, and mineral uptake in maize (Wahid et al., 2007; Turan et al., 2010; Waqas et al., 2017). For drought tolerance, several studies have demonstrated that photorespiration and raffinose (oligosaccharide) metabolism are essential but vary under various conditions (i.e., combined stresses or a single type of stress) (Obata et al., 2015; Sun et al., 2019; Rafique et al., 2020). Additionally, metabolic changes in metabolic profiles in maize results in changes via cell wall remodeling, maintaining metabolic homeostasis, and signaling mechanisms to tolerate multiple stress conditions.

Similarly, the level of reactive oxygen species (ROS) is accompanied by higher CAT, GR, glutathione-S-transferase activities, chlorophyll content, and membrane stability in maize seedlings under stress conditions (Zeid, 2009; Yadav et al., 2018). For instance, higher osmoprotectants are required for stress tolerance under salt stress conditions (Zeid, 2009). Furthermore, heat shock proteins, kinases, phosphatases, and a cascade of metabolic networks are activated under temperature stress, which induces heat tolerance in maize (Gill and Tuteja, 2010; Tiwari and Yadav, 2019). For a particular farming system, climate-smart agronomic techniques include those that improve farmer resilience to climate shocks and/or reduce productivity loss. To lessen the negative impacts of temperature fluctuations, these procedures are becoming more and more crucial. Plants may benefit from a change in planting time by avoiding the temperature extreme period during crucial growth stages by reducing the chance of heat and chilling damage during the silking and grain filling stages, respectively, the change in planting time greatly reduced the output losses (Waqas et al., 2017).

Since plants are easily exposed to various stresses, they have evolved avoidance mechanisms in response to stress. Several studies have observed induced specific morphological changes in stressful environments in maize. For example, the maize flowering stage is vulnerable to temperatures > 30°C; this results in pollen desiccation during anthesis and delays the silking interval, which negatively affects maize productivity (Parent and Tardieu, 2012; Leitner et al., 2014). Stresses initially inhibit cell proliferation, with prolonged exposure preventing cell expansion and mostly speeding up the vegetative growth and completing their life cycle before the onset of temperature stress (Kavar et al., 2008; Masuka et al., 2012; Godínez-Palma et al., 2013). Identifying the metabolic and physiological networks has provided an excellent foundation for adapting to abiotic stress. These physiological networks are controlled by genes and other molecular networks which serve as a great avenue for developing maize lines that are tolerant to wide arrays of abiotic stresses. Moreover, roots also play an essential role in maintaining plant water uptake for effective drought avoidance in maize. Adjustment of the hydraulic root architectural system, soil and water heterogeneity, and transpiration activities during the day can help maize plants endure heat and drought stresses (Leitner et al., 2014). Furthermore, stress-induced signal transduction proteins and enzymes such as APX, SOD, LEA, and heat shock (HSK) proteins are critical for enduring heat and drought stress, especially during grain filling in maize (Hasanuzzaman et al., 2013; Soliman et al., 2018).

## Abiotic stress-responsive gene resources for improving stress tolerance in maize

The sequencing of the maize genome has identified various abiotic-responsive genes and expanded the genomic resources for exploring abiotic stress tolerance in its gene pool. To date, numerous studies have reported transcription factors, signal transduction genes, and several abiotic-stress-responsive *cis*-regulatory elements involved in abiotic stress adaptation.

### Role of transcription factors, *cis*-elements, and signal transduction genes in abiotic stress tolerance

Transcription factors (TFs) and transcription factor binding sites directly affect the transcriptional regulation of plant genes. Several studies have elucidated the roles of TFs in maize adaptation to abiotic stresses. For example, *ZmNF-YB16* has been introduced into the inbred maize line (B104). The overexpression of this TF increased the expression of genes encoding antioxidant enzymes, antioxidant synthase, and molecular chaperones associated with the endoplasmic reticulum stress response. As a result, lines sustained greater photosynthesis, improved dehydration, and drought stress tolerance during vegetative and reproductive stages, increasing maize grain production under drought-stressed conditions (Wang et al., 2018a). Similarly, the WRKY genes in maize have been well associated with abiotic stress resistance. For example, the *ZmWRKY40* and *ZmNF-YB2* gene encoding a transcription factor has improved drought tolerance in maize through functional genomics studies (Nelson et al., 2007; Zhang et al., 2008; Wang et al., 2018b; Gangola and Ramadoss, 2020). The *ABP9* gene in maize encodes the bZIP transcription factor in Arabidopsis, improving salt, drought, and cold tolerance by increasing oxidative enzyme levels (Zong et al., 2018). In addition, several MYB-related proteins in the maize genome have been identified, and 46 of them were already characterized in relation to different abiotic stresses. Among them, the expression of *ZmMYB30* was observed to have increased remarkably under drought and high salinity conditions (Nelson et al., 2007; Wang et al., 2018a; Zong et al., 2018). The MYB TF, *ZmMYB31*, repressed isopalmitate biosynthesis, increased UV exposure sensitivity, decreased plant height, and activated several stress-responsive genes (*Zm5H*, *C3H*, *ZmActin*, and *ZmCOMT*) in transgenic maize plants (Fornalé et al., 2010). Interestingly, *ZmSAPK8*, an *SnRK2* phosphokinase, was cloned from maize that confers salinity tolerance with transcriptional upregulation of stress-linked genes, including *DREB2A*, *P5CS1*, *RAB18*, *RD29A*, and *RD29B* under salt stress indicating *ZmSAPK8* is involved in diverse signal transduction and has the potential to improve salt tolerance in crops (Ying et al., 2011). Other transcription factors like *ZmNF-YB2* and *ZmNAC3* were previously characterized through functional genomics and were described as stress-induced genes in maize that responds to physiological variations in photosynthesis and polysaccharide metabolism (Table 1).

**TABLE 1:** Transcription factors and signal transduction genes associated with abiotic stress tolerance in maize.

Name	Function	Type of stress tolerance	References
ZmNF-YB16	Transcription factor; Promotes the expression of chaperones, antioxidant enzyme capacity, and photosynthesis in maize	Drought stress tolerance	Wang et al., 2018a
ZmNF-YB2	Transcription factor; Promotes the expression of chaperones, antioxidant enzyme capacity, and photosynthesis in maize	Drought stress tolerance	Nelson et al., 2007
ABP9	Transcription factor: Encodes a bZIP transcription factor, binds to the abscisic acid (ABA)-responsive-element (ABRE2) motif of the maize catalase1 gene	Drought stress tolerance	Wang et al., 2017
ZmMYB31	Transcription factor; induces the expression of several stress-responsive proteins.	Oxidative stress modulation	Fornalé et al., 2010
ZmSAPK8	Transcription factor; Essential component possibly through phosphorylation-mediated regulation of downstream substrates	Salinity stress tolerance	Ying et al., 2011
ZmDREB2A	Transcription factor: Regulates genes encoding late embryogenesis abundant (LEA) proteins and genes related to heat shock and detoxification	Water and heat stress tolerance	Qin et al., 2007
Rab17	Plays a specific role in growth inhibition in embryonic tissues, probably in germination and in the induction or maintenance of dormancy of the embryos during desiccation.	Drought stress tolerance	Vilardell et al., 1991
ZmNAC3	Transcription factor; Encodes a nucleus-targeted protein that has an extremely conserved NAC domain in the N-terminus.	Salinity and cold stress tolerance	Li and Jiang, 2021
CAT-1	Cat1 mRNA accumulation may compensate in the absence of other catalases, and that CAT-1 plays a major protective role in response to high temperature stress.	Heat stress tolerance	Scandalios et al., 2000
ZmbZIP72	Transcription factor; Functions as an ABA-dependent transcription factor in positive modulation of abiotic stress tolerance	Salinity and drought stress tolerance	Ying et al., 2011
NPK1	Nicotiana protein kinase/Enhances drought tolerance	Drought stress tolerant	Shou, 2004
ZmATG8	ZmATG genes presented *cis*-regulatory elements involved in osmotic stress response via abscisic acid (ABA)-dependent and ABA-independent signaling	Drought stress tolerant	Tejeda et al., 2019
ZmATG12	ZmATG genes presented *cis*-regulatory elements involved in osmotic stress response via abscisic acid (ABA)-dependent and ABA-independent signaling	Drought stress tolerance	
ZmHsf01	Plays a significant role in heat shock signal transduction and downstream gene expression	Heat stress tolerance	Zhang et al., 2020

Stress can also cause genome-wide transcriptome reprogramming in plants to respond to environmental stimuli. As a result, groups of genes linked to various physiological features and response pathways are controlled to counteract their negative impacts (Shao et al., 2021). Several transcriptomic studies have identified regulatory networks crucial to stress tolerance in maize. For example, a microarray-based technique was used to explore gene expression dynamics throughout seed development, which found 3445 genes with variable expression across samples from six different time points (Liu et al., 2008). Wang B. et al. (2019) demonstrated that drought-induced transcriptomic changes were strongly linked to developmental and physiological adaptation, affecting maize production. Similarly, the transcriptome sequencing of the maize roots grown in N, P, and K deficient environments identified a total of 2555 (N), 2340 (P), and 1173 (K) differentially expressed genes that are involved in nutrient utilization, plant hormones, and transcription factors (Ma et al., 2020).

*Cis*-acting elements function as stress signaling factors at terminal points in signal transduction pathways. In general, they impart several auxiliary functions to the plant systems, such as developmental regulation of growth-associated processes, morphological modifications, regulating senescence, and DNA damage repair mechanisms. Like TFs, *Cis* elements and signal transduction genes were also associated with abiotic stress response in maize. For example, in a recent study, the exogenous application of ABA-induced *CAT1* (*Catalase1*) expression via the *cis*-element *ABRE2* affects the enzymes that are related to ROS (H_2_O_2_) in maize (Guan et al., 2000). Similarly, several ectopic expressions of genes such as *AtNHX1* and *NPK1* were found to regulate transporters, ion channels, and oxidative signaling in response to salinity cold, heat, and salinity tolerance (Yin et al., 2004; Lin et al., 2014). Tejeda et al. (2019) characterized autophagy-related genes (ATG genes; *ZmATG8* and *ZmATG12*) in maize landraces under osmotic stress and found them potential targets for functional characterization and development of osmotic-tolerant maize genotypes using molecular breeding strategies (Table 1).

### Role of micro RNAs in abiotic stress tolerance in maize

MicroRNAs (miRNAs) were also documented to control abiotic stress tolerance in plants by binding to *cis*-regulatory elements or genes/transcripts. For example, miRNAs regulate target gene expression post-transcriptionally and play important roles in seed germination, ear development, and root architecture in maize (Spollen et al., 2008; Peng et al., 2018; Wani et al., 2020). Table 2 shows various miRNAs that are involved in maize abiotic stress tolerance.

**TABLE 2:** Abiotic-stress related miRNAs in maize.

Stress	Plant part	Key	Stage	Regulation	Target genes	References
Submergence	Roots	miR166, miR167, miR171, miR399, osa-miR396-like	Early phase	Up	Transcription factors, including HD-ZIP, auxin response factor, SCL, and WRKY domain protein	Zhang et al., 2008
		miR159, ath-miR395-like, ptc-miR474-like, osa-miR528-like	Early phase	Down	Carbohydrate and energy metabolism, including starch synthase, invertase, malic enzyme, and ATPase	
Salinity	Roots	miR162, miR168 and miR395	–	Up	AGO1, DCL1 for homeostasis and feedback regulation, NADP dependent malic protein, ATP sulfurylase	Ding et al., 2019
		miR156, miR164, miR167, miR396	–	Down	NAC1, ARF8, R2R3 MYBSBP-domain protein, cytochrome oxidase	
Salinity and drought	Seedlings	miR156, miR164, miR166, miR168, miR171, miR319	–	Both	AGO1, leaf and shoot development, hormone signaling	Kong et al., 2010
Drought	–	miR1, miR3, miR6, miR479, miR782, miR815a, miR820	–	Both	Signal transduction, transcription regulation, and biotic or abiotic stress responses	Xu et al., 2010
Drought	Seedlings	miR156, miR159, miR319	–	Up	SPL6, SPL7 and SPL11, MYB33 and MYB101, TCP transcription factors	Li et al., 2013
Drought	Seedlings	miR159, miR168	–	Up	Transcription factor MYB55, Argonaute 1	Wang et al., 2014
Drought, hormone and salinity	Seedlings	miR169	Short term	Down	ZmNF-YA14	Luan et al., 2014
			Long term	Up		
Drought	Seedlings	miR156, miR159, miR160, miR169, miR166, miR393, miR395	–	Up	SPL, MYB, ARF, NFY-A, HD-ZIPIII, TIR, APS/AST	Aravind et al., 2017
Submergence	Seedlings	miR172a, miR164a	–	Down	ERTF-RAP2-7, POD-1	Azahar et al., 2020
Drought	Seedlings Shoot	miR164	–	Down	MYB, NAC	Liu et al., 2019
	Root	miR159, miR390	–	Up	MYB, LRR	
		miR398	–	Down	SPL	
Water deficit	Seedlings	miR399e,i,j-3p	–	Up	ubiquitin conjugating enzymes	Seeve et al., 2019
High temperature	Leaves	miR172a/b	–	Up	AP2/EREBP TF	Zhang et al., 2019b
		miR164, miR169i, miR156a/j/k, miR159, miR166a, miR396a, miR5381, miRn202	–	Down	NAC, SBP, SPL, MYB, HD-ZIP, GFR, VPS24 TFs	
Chilling	Seedlings	miR408, miR528	–	Up	CYCD1/5, GRF1, TCP, ARF, CYCB2	Aydinoglu, 2020
		miR319, miR395	–	Down	GAMYB, CMT	
Submergence and drought	Seedlings	miR156, miR159, miR164, miR166, miR167, miR169, miR396, miR398, miR408,	–	Down	SPL, NAC, GAMYB, GRF, MYB, ARF, NFYA, PLC, LAC, SOD, SBP1, bZIP	Sepúlveda-García et al., 2020

Short tandem target mimics (STTM) is a technology that develops a resource for producing miRNA inactivation vectors and transgenic lines in model and crop plants (Peng et al., 2018). A recent study on a series of maize STTM166 transgenic plants identified 178 differentially expressed genes (60 downregulated and 118 upregulated genes). Most were involved in the cell membrane system, cell components, oxidation-reduction process, oxidoreductase activity, and carbohydrate metabolic processes. Several studies were also carried out to identify novel miRNA and mRNA interaction and their association with abiotic stress tolerance in maize. For example, a microarray expression analysis of a drought-associated study revealed 13 miRNAs families regulating 42 novel mRNAs target and 65 in maize (Li et al., 2020). Another study showed that 23 drought-responsive cis-regulatory elements and three TFs (*GAMYB*, HD-Zip III, and NAC) were associated with the target mRNAs (Aravind et al., 2017; Sepúlveda-García et al., 2020). The knock-down maize mutants of *miR166* showed its association with adaptation through various phenotypic variations. It was observed that knock-down mutants had rolled leaves, inferior yield-related traits, epidermis structures, vascular patterns, enhanced abiotic stress resistance, elevated ABA levels, and reduced levels of indole acetic acid. The results shed light on the importance of ABA and auxin interaction in monocots and suggested that the specific mechanism differs from dicots (Aydinoglu, 2020; Li et al., 2020).

Du et al. (2016) performed overexpression studies to understand and improve the adaptive response of phosphorus (Pi) deficiency in maize and found a phosphorus-deficiency-induced long non-coding RNA1 (*PILNCR1*) that inhibits *miR399* and thus *miR399*-mediated cleavage of *ZmPHO2* [*Zea mays* PHOSPHATE2 (*PHO2*) pathway]. These findings indicate interactions among PILNCR1 and miR399 that could be potential targets for improving phosphorus efficiency in maize. Maize mutant lines for *miR166* and its target gene *Rld1* are involved in Rolled leaf 1 (*Rld1*) (a homologous gene of Arabidopsis HD-ZIP III transcription factor). The interaction of these two components is responsible for leaf polarity and exhibits various developmental defects such as delayed flowering, reduced stature, and curled leaf. Other mutants related to leaf polarity have been documented, including leaf-bladeless1 (*lbl1*), Arabidopsis SGS3 homolog, ragged seedling2 (*rgd2*), Arabidopsis *ago7* homolog, *milkweed pod1* (*mwp1*), and Arabidopsis *KANADI* homolog (Juarez et al., 2004; Douglas et al., 2010). Intriguingly, *miR390-lbl1* and *miR166-rld1* have been implicated synthesis, transport, and action in maintaining leaf developmental patterns (Nogueira et al., 2007, 2009; Itoh et al., 2008).

### Quantitative trait loci and genome-wide associated studies for abiotic stress tolerance in maize

The current advances in sequencing have led to the development of genetic tools to understand complex traits in crops. Over the years, these tools were successfully used to develop new genetic resources that could be used to mine beneficial genes and marker-assisted breeding (Kitony et al., 2021; Reyes et al., 2021b). Investigating complex traits like abiotic stresses requires quantitative genetic approaches such as linkage mapping and association analysis using different mapping populations or association panels (Almeida et al., 2013; Samantara et al., 2021).

To date, QTL governing various abiotic stresses have been mapped using backcrossing, F_2_, RILs, introgression lines, and natural populations (Qiu et al., 2007; Mano and Omori, 2008; Osman et al., 2013; Zaidi et al., 2015; Frey et al., 2016). For cold tolerance, Hund et al. (2011) mapped important putative meta-QTL (*MQTL Rt-6*, *MQTL Ax-2*, *MQTL Rt-7*, and *MQTL Ax-15*) for root architecture traits during cold stress. Similarly, Hu et al. (2016) identified 12 QTL for germination rate and primary root length in maize under cold stress using B73 × Mo17 (IBM) derived population. Interestingly, the candidate gene GRMZM2G398807 governs cortical cell-delineating protein expression in the primary root, assisting in radicle protrusion. Goering et al. (2021) identified two QTL with high additive impact on chromosomes 1 and 4 using a panel of IBM Syn4 recombinant inbred lines. The genes with putative function related to auxin and gibberellin response were identified in the QTL region. Recently, Han et al. (2022) mapped five QTL clusters on chromosomes 1, 2, 3, 4, and 9 using an B73 x Mo17 (IBM) Syn10 double haploid population. From these clusters, 39 genes were extracted, and through RNA-seq, upregulated genes from B73 and Mo17 were identified.

For heat stress, Frey et al. (2016) identified two significant QTL for grain yield and one QTL for the leaf scorching trait. Similarly, McNellie et al. (2018) identified three major QTL, of which two major QTL were mapped on chromosome 3 (for plant death and leaf firing) and chromosome 1 (for leaf firing) in B73 × CML103 under heat stress at the late vegetative stage. The co-localized QTL for plant death and leaf firing reveals chromosome 3 as a potential region for heat stress tolerance. Inghelandt et al. (2019) identified QTL related to heat susceptibility index (HIS) of five traits on chromosome 2, 5, 9, and 10 using dent and flint maize inbred line populations. The study revealed an antagonistic pleiotropy between heat tolerance at seedling stage and adult stage. However, a low PVE was observed in these QTL making it not suitable for marker-assisted breeding applications.

Quantitative trait loci mapping under drought condition is associated with multiple traits such as anthesis–silking interval (ASI), root architecture, and grain yield traits. Giuliani et al. (2005) elucidated the role of *Root-ABA1* on root architecture and leaf ABA concentration in response to different water deficit conditions. And in Landi et al. (2006) further validated the effect of this QTL under two environments (China and Italy). Almeida et al. (2013) mapped two significant constitutive QTL (chromosomes 2 and 6) and two adaptive QTL (chromosomes 5 and 7) for grain yield under drought stress. Semagn et al. (2013) identified four meta-QTL (*mQTL2.2*, *mQTL6.1*, *mQTL7.5*, and *mQTL9.2*) for grain yield under water-deficit and well-watered conditions. Zhao et al. (2019) identified three major QTL for drought stress tolerance (*qKR-Ch.1-1* for kernel ratio, *qEHPH-Ch.1-1* for ear height to plant height ratio, *qGW-J1-1* for grain weight per ear, and *qGW-Ch.4-1* for grain weight per ear) that could be targeted for introgression. Similarly, genetic regions that are associated with plant height, root length, and dry weight traits under salinity stress were mapped in the maize genome (Qiu et al., 2007; Osman et al., 2013).

Genome-wide associated studies (GWAS) is a powerful tool for dissecting genetic loci that significantly influence agronomic traits based on distinct phenotypes and extensive coverage of molecular markers. Many initiatives related to abiotic stress that include stress-linked genes in agricultural plants have used genome-wide association studies. For example, Liu et al. (2013) conducted GWAS using 368 maize varieties at the seedling stage and identified connections between the genetic variation in *ZmDREB2.7* and the degree of drought tolerance in different maize varieties is highly dependent on DNA polymorphisms in the promoter region of *ZmDREB2.7*. In a study conducted by Thatcher et al. (2016), the genome-wide analysis showed significant changes in ears and leaves, magnifying that developmental splicing is linked to developmental stage, tissue type, and stress conditions. Song et al. (2016) identified cysteine-rich poly-comb-like (CPP) proteins in maize, and different expression levels of the *ZmCPP* gene under abiotic stresses (cold, salt, heat, and drought), indicated that *ZmCPP* functional characterization in maize could help understand maize growth and development. Using a genome-wide analysis of the *AP2/ERF* TF family, Zhou et al. (2012) showed that *AP2/ERF* gene family could participate in various stress responses such as drought and salinity. The same approach was carried out by Luo et al. (2019), wherein two promising genes, *SAG4* and *SAG6* (salt-tolerance-associated gene), were identified and have a great potential to develop salt-tolerant maize lines. Similarly, the GWAS approach was used to identify genetic regions associated with root-related traits, drought tolerance, and nitrogen use in maize (Wang N. et al., 2019; Guo et al., 2020). The success in GWAS utilization showed its great importance for studying multiple traits under drought, salt, and temperature stresses. GWAS studies in maize have identified novel gene candidates or genes responsible for abiotic stress, which can be used for maize breeding and designing climate-smart maize lines.

## Breeding approaches for improving abiotic stress tolerance

### Utilization of maize wild relatives as a genetic resource

Maize (family Poaceae) comprises seven genera, including *Chionachne, Sclerachne, Coix, Trilobachne, Polytoca, Tripsacum*, and Zea. The genus *Zea* contains four species, but only Zea mays L. (2n = 20) is economically valuable. The other *Zea* species, referred to as teosintes, are essentially wild grasses native to Mexico and Central America (Doebley, 1990). The origin of maize (*Zea mays* L.) dates back more than 7,000 years and seems to have developed from annual teosinte (*Zea mexicana*) through gradual selection. The wild ancestor of modern maize has comparable plant architecture and growth forms to maize, while *Tripsacum* has higher chromosome numbers (2n = 36, 64, or 72), making it more challenging to hybridize with maize. These wild ancestors of maize have considerable genetic diversity and could be a new resource for germplasm enhancement (Maazou et al., 2016). For example, molecular evolution studies on maize have included genetic and genomic tools developed for Tripsacum (Blakey et al., 2007). Data from orthologous genes in maize and the *Tripsacum* genus revealed a distinct collection of genes with frequent non-synonymous substitutions in *Tripsacum*, which were prevalent when domestic maize was adapted to temperate regions through artificial selection. An intermediate metabolic route, phospholipid metabolism, was linked to cold and freezing tolerance. Anatomical descriptions such as aerenchyma tissue in roots and other properties in *Tripsacum* have contributed to survival under drought stress. Similar studies on root penetration and increased biomass in *Tripsacum* have revealed drought resistance. Physiological data revealed that the exceptional drought resistance of the *Tripsacum* genus is due to its high-water use efficiency and photosynthetic levels (Kemper et al., 1998). Later research revealed that the *Tripsacum*-introgressed cultivar grows better than modern maize under drought stress. *Tripsacum* introgression lines appear to have better-rooting systems that penetrate deeper into the soil and higher grain yields than modern maize cultivars (Gilker et al., 2002; Eubanks, 2006; Gitz et al., 2013). An evaluation of maize (*Zea mays* ssp. mays) × *Tripsacum dactyloides* L. hybrid (eastern gamagrass) calli revealed that hybrid plantlets had higher fresh weight than maize plants under salt stress and thus improved salt tolerance (Shavrukov and Sokolov, 2015). These hybrids retain sodium in their leaves, decreasing water potential and maintaining turgor pressure, vital for vegetative development (Pesqueira et al., 2003). Therefore, tapping the genetic potential of wild relatives is necessary to create new genetic resources that can serve as future accessions to develop maize lines with improved abiotic stress tolerance.

### Speed breeding and genomic selection

Traditional maize breeding can be used to develop high-yielding hybrids from selective parental mating based on specific combining abilities. The development of parental lines require 4–6 years and another 1–2 years for their hybrids, resulting in a long and somewhat complicated breeding cycle. To address this, speed breeding approaches were introduced (Hickey et al., 2017; Samantara et al., 2021). Several approaches can be used to shorten the breeding cycle in maize including (a) off-season nurseries, (b) double haploid (DH) technology, and (c) *in vitro* nurseries. The off-season nursery approach grows crops in a different location suited to their photoperiodism. DHs can be produced in two generations, significantly reducing the breeding cycle (Geiger and Gordillo, 2009). The most common method for maize DH uses the R1-nj color marker. *In vitro* nurseries are another option for breeding homozygous and homogenous lines, with a mix of DH technology (homozygosity per generation) and off-season nurseries (generations per year). These methods have several advantages and disadvantages. Off-season nurseries require quarantine clearance or other permissions to grow crops in a different location, extra time to transport genetic material and could introduce new pests and diseases. DH technology can have low success rates, hindering application for haploid induction and tissue culture adaptation. In addition, doubling is genotypic dependent and uses a carcinogenic agent (colchicine).

**FIGURE 4:**
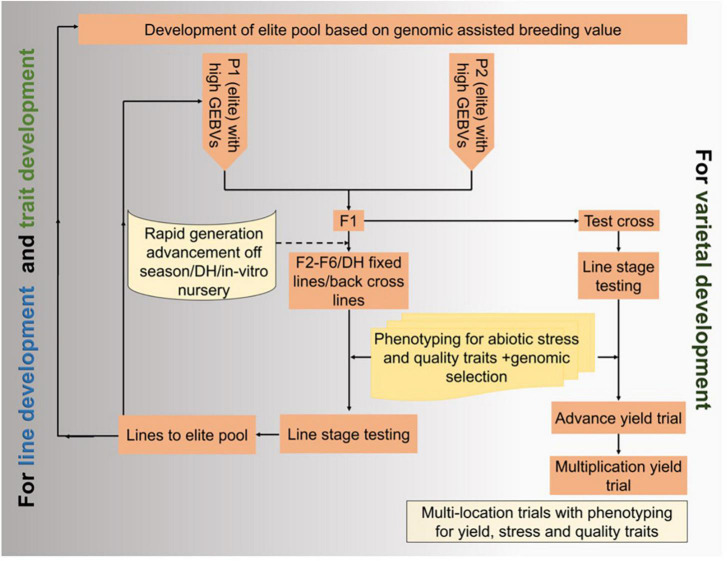
Integration of speed breeding and genomic selection in maize cultivar improvement and development.

Genomic selection helps accumulate favorable genes with minor effects and improves multigenic traits, especially in stress-prone environments with a high environmental variation. The genomic selection focuses on estimating breeding values using many molecular markers that ideally cover the entire genome to predict the genetic value of the candidate for selection and using genetically estimated breeding values (GEBVs) to advance populations through rapid-cycle genomic selection without phenotyping in each cycle (Massman et al., 2013). Figure 4 is a schematic diagram integrating genomic selection and speed breeding for varietal and line development.

Genomic selection improves polygenic traits, governed by small-effect genetic loci, such as plant yield. This tool uses marker effects across the genome to estimate GEBVs. Genomic selection produced higher stover and grain yield than MAS in a bi-parental maize population (Massman et al., 2013). Similarly, two fast cycles each year via genomic selection increased grain production by about 2% in a multi-parental maize population (Zhang et al., 2016). The accuracy of genomic predictions correlated with the test cross’s marker-predicted genotypic and phenotypic values in the validation population. The model’s genomic selection prediction accuracy was more accurate than genomic selection and marker-assisted selection. It could be used to capture alleles with fewer additive effects to increase genetic gain under drought. The predicted results differed slightly between the training and validation sets or linkage disequilibrium with causal variants in another study. The mean performance of the breeding populations differed from the underlying predicted traits (Windhausen et al., 2012). Genomics selection combined with MAS (marker-assisted selection, GS-MAS) had advantages over the QTL-MAS approach for drought-stressed growth characteristics (grain yield, anthesis, and plant height), evident from the differences between R2 (QTL-MAS) and prediction accuracies (GS-MAS) in the analysis (Tilman et al., 2011). Combining molecular marker data and phenotypic data as input variables delivered higher-quality estimates for grain production on average than phenotypic data alone (Trachsel et al., 2019).

Maize is susceptible to abiotic stresses, especially drought and waterlogging, allowing a higher degree of genotype × environmental interaction in a breeding program. To identify those with higher yield potential in the target environment, maize lines/hybrids were evaluated under targeted or managed stress conditions. However, most of the parents/hybrids/crosses were discarded due to poor performance in the field, which could be due to the low genetic value of the parents selected for crossing. Thus, parents of high GEBV should be used for crossing to accumulate the maximum number of favorable alleles for desirable traits in the progenies. Further, genetic gain is limited under abiotic stresses and high environmental error (Trachsel et al., 2019). Thus, combining genomic selection and speed breeding for abiotic stress tolerance would enhance the selection accuracy of parents to increase genetic gain and abiotic stress tolerance.

### Genome editing

Genome editing tools are some of the major breakthroughs in molecular biology. These techniques offer a precise and efficient way to edit an organism’s genome. Some of the well-known genome editing tools are ZFNs (zinc-finger nucleases), TALENs (transcription activator-like effector nucleases), and clustered CRISPR (regularly interspaced short palindromic repeats)/Cas systems (Miller et al., 1985; Jinek et al., 2012; Joung and Sander, 2013). These tools use common sequence-specific nucleases to identify specific DNA sequences in the genome and generate desired double-stranded breaks (DSBs) (Mushtaq et al., 2021). ZFNs have been used to modify various crop plants, including maize. The ZFN-mediated targeted transgene integration was used to stack characteristic traits, especially for stress tolerance, in maize by combining several beneficial features (Ainley et al., 2013; Mushtaq et al., 2021). On the other hand, TALENs are a promising genetic tool for targeted gene mutagenesis in maize and other crops. This approach has been used to produce stable mutations at the maize glossy2 (*gl2*) locus that are heritable. The transgenic maize lines with mono-di-tri- allelic mutations conferred the glossy phenotype (Char et al., 2015). In most cases, integrated TALEN T-DNA separated independently from the new loss of function alleles, giving rise to null-segregant offspring in the T1 generation; thus, TALENs are an efficient tool for maize genome mutagenesis for identifying gene function and improving abiotic stress-related characteristics. Constructing TALEN repetitions is complex and TALEN gene targeting effectiveness varies.

CRISPR/Cas is a flexible genome editing technology with potential applications. Compared with ZFNs and TALENs, the CRISPR/Cas system has been adopted rapidly in plants due to its high efficiency, simplicity, cost-effectiveness, and ability to target multiple genes (Cong et al., 2013; Hsu et al., 2014; Razzaq et al., 2019). Several abiotic stress tolerance studies have been carried out using CRISPR/Cas. For example, drought-resistant characteristics have been incorporated into maize using CRISPR/Cas9 gene insertion and substitution techniques. To explore the targeted use of ARGOS8 native expression variation in drought tolerance breeding, CRISPR-Cas was used to generate novel variants of ARGOS8 (Shi et al., 2017). The low-expression gene ARGOS8 in maize, which negatively regulates ethylene responses, is crucial for plant growth. Field evaluation of lines showed that the ARGOS8 variations produced five extra bushels of grain production per acre under drought stress compared to the wild type. These findings show that CRISPR-Cas9 technology can create new allelic diversity in crops by substituting breeding drought-tolerant crops. In another study, CRISPR/Cas9 was used to unravel the abilities of the *ZmWRKY40* gene encoding a transcription factor (Wang et al., 2018b), which improved drought tolerance, and resultant lines imparted drought tolerance in maize.

Therefore, genome editing techniques have a great potential to improve crops. After carefully selecting the genome-editing tool, target sequences can be designed and introduced into the most appropriate vectors. Suitable genetic cargo (DNA, RNA, or RNPs) is then selected for delivery by (i) modifying the targeted sequence, (ii) regenerating the edited calli, and (iii) producing the edited plants. Combining earlier genome editing tools with newly developed breakthroughs is speeding up genome editing in crop breeding to meet the world’s exponentially increasing need for food. In addition, climate change necessitates flexibility and ingenuity regarding crop resilience and production methods. Moreover, we must consider government restrictions and the public acceptability of these new breeding methods.

### Metabolomics

Numerous research digging into its systems biology, maize has benefited from the introduction of omics techniques. Because maize and its derivatives are used as both food and bioethanol, the metabolism of maize has received significant study. As reviewed by Medeiros et al. (2021) several aspects of maize metabolism have received significant attention, including (1) the role of the metabolome in basic molecular processes, responses to biotic and abiotic stresses, and beneficial biotic interactions; (2) the nutritional composition of maize kernels and the molecular mechanisms that underpin the production of specific metabolites; and (3) the mechanisms by which the metabolome and metabolic models link to leaf physiology. (4) The metabolic changes caused by genetic modifications; and (5) the degree of natural metabolic variance and its potential utility in breeding efforts.

In a study by Alvarez et al. (2008), changes in stomatal hormones like as ABA and cytokinins were discovered, underlining the importance of root-to-shoot signaling in maize under drought condition. Furthermore, an increase in the content of phenylpropanoid pathway intermediates was found. In a study by Casati et al. (2011), metabolomics was used to document the candidate signaling molecules associated with temporal effects of irradiation of canopy leaves in maize. The study identified myoinositol as a candidate molecule for UV-B responses. Witt et al. (2012) used the same approach to assess metabolome changes under drought condition. However, differential changes were not detected in their experiment which could possibly be attributed to experimental design such as the use of greenhouse instead of actual field setup. Metabolite profiling under salt stress conditions was carried out by Richter et al. (2015). Using two differently resistant maize hybrids, they were able to deduce that TCA cycle and sugar metabolism are highly affected biochemical pathways during salt stress. Sun et al. (2016) investigated the metabolic growth and response of maize to cyclic drought using a metabolomic approach. Their study demonstrated that distinct metabolic pathways in maize plants returned to normal at different speeds during recovery. However, metabolic study revealed quantitative differences between the two cycles, indicating the intricacy of metabolic processes launched by water cycle alterations.

Other than pathways, metabolomic studies have aided in showing which organs in maize are mostly affected by abiotic stress. For example, during drought condition, leaf blades were identified to have the greatest metabolic changes (Witt et al., 2012; Obata et al., 2015). And under high-salt conditions, metabolic changes were found to be greater in shoots than in roots (Gavaghan et al., 2011). In a study conducted by Ganie et al. (2015), metabolomic approach highlighted that under phosphorus starvation conditions, leaves are the main site for metabolic changes.

### High throughput phenotyping

Conventional phenotyping uses destructive approaches to measure drought, heat, and salt tolerance characteristics. However, the development of high throughput phenotyping (HTP) platforms has greatly reduced the phenotyping issues that limit breeding programs. To date, several high-throughput phenotyping methods have been used to characterize maize phenomes, including penetrometers, electrical conductivity sensors to assess soil mapping variability, thermal imaging to monitor plant canopies, and spectral reflectance (Masuka et al., 2012). In addition, satellite imagery, mobile cameras, UAV imaging, and ground-based imaging platforms are widely used in maize plant stress assessment.

Typically, high-throughput plant phenotyping is accomplished by collecting photos that quantify attributes across a crop plant’s whole life cycle as traditional phenotyping is inefficient, expensive, and inaccurate (Arya et al., 2022). Nowadays, satellite imaging technology has become a powerful tool for collecting data in large agricultural practices, but few apply to small experimental breeding plots. For instance, satellite-driven NDVI (normalized differential vegetation index) strongly correlated with UAS multispectral imagery in maize plots (24 m2) (Sankaran et al., 2020). Unmanned aerial vehicle-red, green, and blue (UAV-RGB) images supplemented UAV thermal images for precise extraction of maize canopy temperature (Zhang et al., 2019a). In addition to general data measurement methods, advanced tools such as digital imagery, stable isotopes, spectral reflectance, and thermal imagery are used to improve data accuracy for soil and climate measurements. Maize studies have used SPAD meter, NDVI, and infrared gas analyzer (IRGA) to quantify environmental effects, digital imagery to measure early biomass in response to water stress, and thermal imagery to quantify leaf temperature during transpiration (Marti et al., 2007; Jones et al., 2009; Rorie et al., 2011). In several studies, infrared thermography (IRT) has been used to measure temperature differences between leaf, air, and canopies under heat and drought stress, spectroscopy to monitor the photosynthetic rate of leaves and canopies in response to the early onset of water stress, and fluorescence imaging to scrutinize plant growth during drought and low-temperature stress (Zia et al., 2013; Lee et al., 2015; Boote et al., 2016). Recently, fluorescence imaging was used to investigate plant chemical composition via spectral absorption and reflectance from cell to canopy level (Zhang et al., 2019a). The multispectral imaging techniques like X-ray-CT, MRI, and ultraviolet spectra (UV) can report changes in ionic balance, stomatal conductance, and transpiration, contributing to drought and high-temperature stress resistance in maize (Huang et al., 2016).

The fast-paced development of these HTPs have generated large number of datasets. And to date, one of the major challenges is the analytical approaches for these datasets. As previously reviewed, machine learning and deep learning techniques are some of the emerging techniques that can be used in the identification of hidden relationships between large datasets (Singh et al., 2018, 2021; Arya et al., 2022; Gill et al., 2022). In a review presented by Singh et al. (2018), both ML and DL are identified to be seamlessly integrated into data acquisition, data preprocessing, and data analytics for real-time HTP of plant traits in the field. Recently, Gill et al. (2022) highlighted that the current ML-based techniques focuses on a single stress or disease on a leaf or canopy, but in real-world situations, numerous diseases and stresses may appear on a single leaf or canopy. Therefore, ML platforms must be flexible and robust, with the ability to distinguish multiple disease symptoms on a single leaf or within the same plant canopy. Collectively, the quantity of data needed to train ML models is determined by the complexity of the problem and the complexity of the learning algorithm; thus, for wider applicability, the training data should really be continuously updated using techniques such as artificial learning to reflect the complexity of stress symptoms for the targeted crop.

## Conclusion

This review concludes that the utilization of omics approaches is necessary to improve the abiotic stress tolerance in maize. The advancement in data utilization from high-throughput sequencing and phenotyping is necessary to exploit their potential in crop improvement fully. In addition studies on stress-tolerance-related mechanisms should consider the stress duration, which affects molecular and physiological reactions. However, short-term solutions are a more common practice among researchers. The molecular foundation of plant stress tolerance is influenced by stresses that occur under natural circumstances throughout the seasons. Thus, comparative studies on the expression and function of gene families under extreme conditions will help reduce the impact of abiotic stress in maize.

An opportunity for advancement in the omics approaches is also necessary. For example, integration of various omics approaches and *in silico* modeling can be employed to further dissect the genetics of abiotic stress tolerance in maize. These will widen our knowledge of molecular cascades and intracellular mechanisms governing stress adaptation. In addition, several modifications on the analytical approaches for the data generated can be implemented. For instance, HTS generates large amount of data. However, a bias in terms of analysis serves as a bottle neck to fully utilize the data. Using computer science and engineering, bioinformatics pipeline can be further fine-tuned to be more fitted for future analytical use. The same is true with HTPs, further refinement in the DL and ML algorithms is necessary to cater the needs of complex analyses.

## Author contributions

SW, MF, and KHMS conceptualized the manuscript. MF, GN, SW, JC, MR, RS, MA, ZZ, SG, AR, VPR, EM, TE-A, HE, and KHMS contributed to the sections of the manuscript. VPR, MF, and SW contributed to the editing and revising of the manuscript. HE, SW, and KHMS supervised the writing and editing of the manuscript. All authors contributed to the article and approved the submitted version.
